# In the Rat Hippocampus, Pilocarpine-Induced Status Epilepticus Is Associated with Reactive Glia and Concomitant Increased Expression of CD31, PDGFRβ, and Collagen IV in Endothelial Cells and Pericytes of the Blood–Brain Barrier

**DOI:** 10.3390/ijms25031693

**Published:** 2024-01-30

**Authors:** Grigorios Kyriatzis, Anne Bernard, Angélique Bôle, Michel Khrestchatisky, Lotfi Ferhat

**Affiliations:** Aix-Marseille Univ, CNRS, INP, Inst Neurophysiopathol, Marseille, France, Institut de Neurophysiopathologie, Faculté de Médecine, 27 Bd Jean Moulin, 13005 Marseille, France; g.kyriatzis@gmail.com (G.K.); anne.bernard@univ-amu.fr (A.B.); angelique.bole@univ-amu.fr (A.B.)

**Keywords:** temporal lobe epilepsy, pilocarpine, hippocampus, epileptogenesis, gliosis, inflammation, blood–brain barrier, endothelial cells, pericytes, basement membrane

## Abstract

In humans and animal models, temporal lobe epilepsy (TLE) is associated with reorganization of hippocampal neuronal networks, gliosis, neuroinflammation, and loss of integrity of the blood–brain barrier (BBB). More than 30% of epilepsies remain intractable, and characterization of the molecular mechanisms involved in BBB dysfunction is essential to the identification of new therapeutic strategies. In this work, we induced status epilepticus in rats through injection of the proconvulsant drug pilocarpine, which leads to TLE. Using RT-qPCR, double immunohistochemistry, and confocal imaging, we studied the regulation of reactive glia and vascular markers at different time points of epileptogenesis (latent phase—3, 7, and 14 days; chronic phase—1 and 3 months). In the hippocampus, increased expression of mRNA encoding the glial proteins GFAP and Iba1 confirmed neuroinflammatory status. We report for the first time the concomitant induction of the specific proteins CD31, PDGFRβ, and ColIV—which peak at the same time points as inflammation—in the endothelial cells, pericytes, and basement membrane of the BBB. The altered expression of these proteins occurs early in TLE, during the latent phase, suggesting that they could be associated with the early rupture and pathogenicity of the BBB that will contribute to the chronic phase of epilepsy.

## 1. Introduction 

Epilepsy is the third most common neurological disorder, affecting more than 65 million people worldwide [1]. Despite progress in anti-epileptic treatments, up to 40% of epileptic patients remain resistant to all currently available therapies, and patients who respond to anti-epileptic treatments often complain of debilitating side effects [2]. TLE is the most prevalent form of epilepsy, particularly affecting the hippocampus. It is characterized by the loss of principal neuronal cells and interneurons, structural reorganization such as sprouting, neo-spinogenesis, and neurogenesis with cell dispersion. TLE is also associated with gliosis, neuroinflammation, and loss of the integrity of the BBB [3]. Each one of these characteristics has been observed in surgical or post-mortem samples obtained from patients with pharmaco-resistant TLE, or from animal models of TLE [3,4,5]. The aforementioned histopathological alterations are thought to take place following an initial injury and contribute to epileptogenesis [5,6]. 

The BBB is a cellular barrier made up of endothelial cells that line brain capillaries and that are encased in a basement membrane (BM)—or basal lamina—in interaction with pericytes, which are closely linked to brain parenchyma cells such as astrocytes, microglia, and neurons [7]. Many proteins are involved in BBB function in physiopathological conditions, which justifies the study of their regulation during epileptogenesis. From these proteins, we selected those involved in vascular and BBB maintenance and expressed in specific cell types of the BBB. Cluster of differentiation 31 (CD31), also known as platelet endothelial cell adhesion molecule-1 (PECAM-1), is expressed mainly by endothelial cells [8,9,10]. Platelet-derived growth factor receptor beta (PDGFRβ) is specific of pericytes, contractile cells that surround endothelial cells and that control blood flow [11,12,13]. PDGFRβ is a biomarker of fibrotic modifications and stromal cell occupancy associated with scar formation [14,15]. We also followed the regulation of collagen IV (ColIV). It is the most abundant component of the BM, and is produced predominantly by endothelial cells, pericytes, and astrocytes, other major constitutive cells of the BBB [16]. Astrocytes interact with, and surround, endothelial cells and pericytes, via specialized structures known as astrocytic end-feet. 

The BBB protects the central nervous system (CNS) very efficiently. It is a selective filter between blood and brain parenchyma, and it regulates the selective uptake of nutrients and proteins from the blood, the influx into the CNS of toxic xenobiotics and pathogens, and the efflux of metabolic waste. BBB integrity is essential for CNS homeostasis, and many disorders of the CNS—including stroke, traumatic brain injuries, tumors, infectious diseases, neurodegenerative diseases, and epilepsy—entail BBB dysfunction [17]. BBB permeability or leakage is one of the earliest characteristics in these diseases [18,19]. BBB dysfunction may contribute to epileptogenesis via a cascade of events triggered by leakage of inflammatory mediators into the CNS, which causes neuroinflammation [20,21]. Conversely, seizures may further increase BBB breakdown [22,23,24]. Alterations of different neuronal and glial populations of the brain parenchyma and their interactions have been extensively studied in epilepsy [25,26]. However, considerably less attention has been given to the regulation of molecular components involved in the integrity and stabilization of the BBB, particularly in regard to TLE. 

In the present work, we injected rats with the proconvulsive drug pilocarpine to induce status epilepticus (PILO-SE), which leads to TLE. We investigated the modulation of reactive glia and vascular markers at several time points of epileptogenesis (latent phase—3, 7, and 14 days; chronic phase—1 and 3 months, abbreviated as PILO 3-14D; PILO 1M; and PILO 3M respectively) using RT-qPCR, double immunohistochemistry, and confocal imaging. We report in the hippocampi of PILO-SE rats the increased expression of mRNA encoding the neuroinflammatory glial proteins GFAP and Iba1, and the concomitant induction of the specific proteins CD31, PDGFRβ, and ColIV in the endothelial cells, pericytes, and BM of the BBB, respectively. These alterations could be associated with the rupture and pathogenicity of the BBB in TLE. Perivascular inflammation of reactive astrocytes and their end-feet in the PILO rat model was addressed previously [27] and was not addressed in the current study. 

## 2. Results

**Figure 1:**
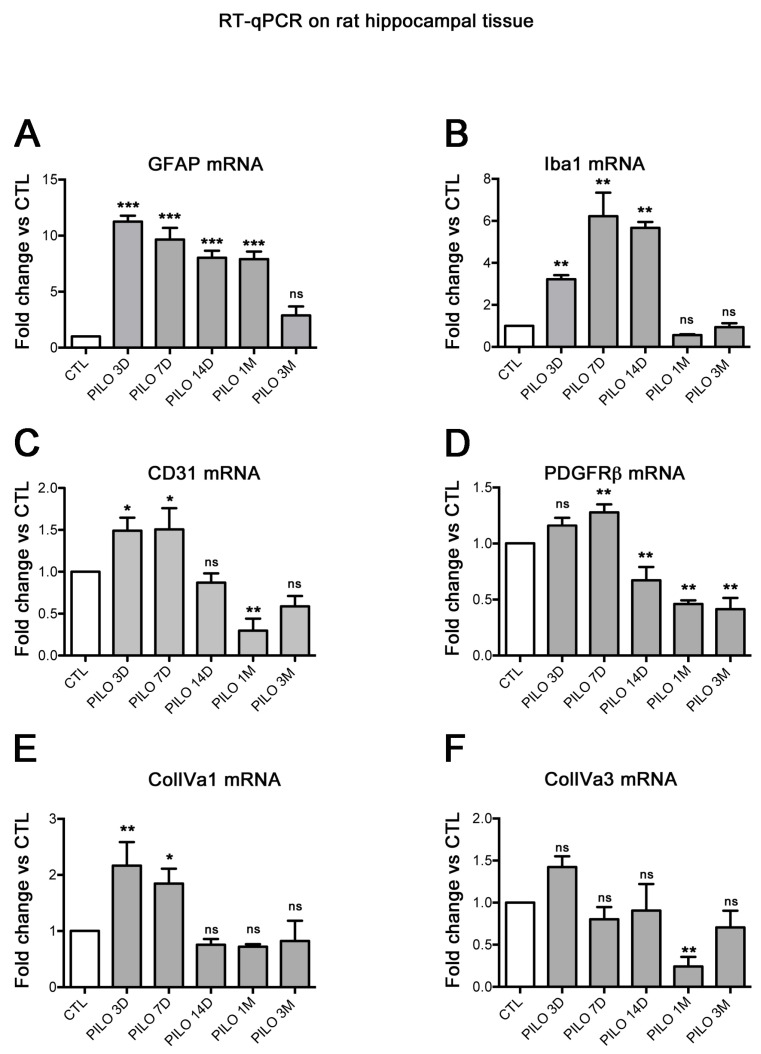
Histograms showing RT-qPCR quantification in rat hippocampal tissue of the mean levels of mRNAs encoding the reactive glial markers GFAP (**A**) and Iba1 (**B**), and the vascular markers CD31 (**C**), PDGFRβ (**D**), ColIVa1 (**E**), and ColIVa3 (**F**) at different time points after PILO-SE. GFAP and Iba1 mRNA were increased at early time points (latent phase, PILO 3D–14D) and decreased during the chronic phase (PILO 1M and 3M). The expression of CD31, PDGFRβ, and ColIVa1 mRNA follows the same trend as the glial markers. ColIVa3 mRNA levels were unchanged at early time points after PILO-SE but were decreased during the chronic phase at PILO 1M. Values are given as the mean ± SEM normalized to CTL. Asterisks indicate statistically significant differences: * *p* < 0.05, ** *p* < 0.01, *** *p* < 0.001 (one-way ANOVA followed by Dunnett’s post hoc test); ns: not significant; in each histogram, we compared the means of controls to the means of each experimental condition; n = 3 rats for each time point.

All PILO rats that survived developed SE. From 10 min to 1 h following PILO injection, rats exhibited limbic motor seizures every few minutes. At 3 weeks, spontaneous recurrent seizures (SRSs) started to appear that could last up to 60 s, and these developed into generalized seizures within the following days, persisting for the lifetime of the animals, in agreement with previous reports [27,28,29,30,31]. We used 2 semi-quantitative approaches to follow the expression and regulation of BBB markers and/or cellular expression and distribution. First, we used RT-qPCR to study global changes of neuroinflammatory and BBB biomarkers at the level of the whole hippocampus. Second, we correlated these results with qualitative and semi-quantitative IHC regarding BBB biomarkers at the cellular level. We thus studied, at different time points after PILO-induced SE, the expression of mRNAs encoding different neuroinflammatory and BBB markers using RT-qPCR (Figure 1) and specific primers listed in Table 1. In particular, we assessed the inflammatory and reactive glial markers GFAP and Iba1, and the vascular markers CD31, specific to endothelial cells; PDGFRβ, specific to pericytes; and ColIVa1 and ColIVa3, specific to the BBB basal lamina [32]. GFAP and Iba1 mRNA increased during the latent phase (PILO 3 to 14D) and decreased during the chronic phase (PILO 1M and 3M). The mean GFAP and Iba1 mRNA levels were significantly increased in PILO rats when compared with control (CTL) rats (*p* < 0.01; Dunnett’s test). Specifically, in PILO 3D, increases were ~11- and ~3-fold for GFAP and Iba1, respectively. In PILO 7D, GFAP and Iba1 increased ~9.7- and ~6-fold, respectively, whereas in PILO 14D, GFAP and Iba1 increased ~8- and ~5.7-fold, respectively. In PILO 1M, GFAP increased ~8-fold, whereas no difference was found for GFAP in PILO 3M or for Iba1 in PILO 1M and 3M (see histograms A and B, Figure 1). It is known that neuroinflammation may lead to alterations of the BBB [4,33]. Consistently, our data show that the upregulation of GFAP and Iba1 mRNA is associated with the significant increase of mRNAs encoding CD31 (PILO 3D and 7D, ~1.5-fold), PDGFRβ (PILO 7D, ~1.3-fold), and ColIVa1 (PILO 3D: ~2.2-fold; 7D: 1.8-fold). This upregulation follows the same temporal course as that of reactive glial markers, although at different levels, suggesting that CD31, PDGFRβ, and ColIVa3 expression is associated with inflammatory processes at the BBB.

**Figure 2:**
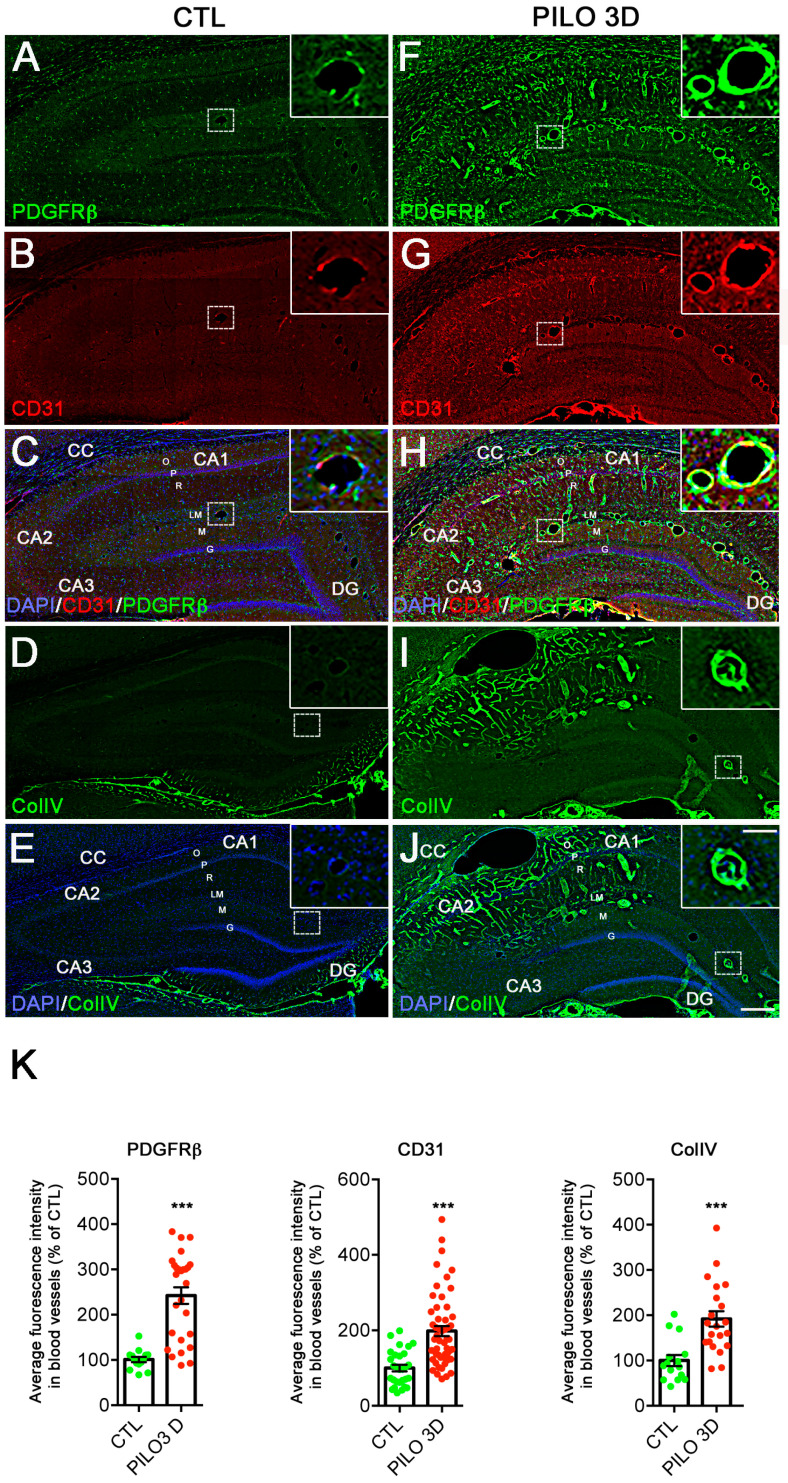
PDGFRβ, CD31 and ColIV protein expression increased in rat hippocampi following PILO-SE. (**A**–**J**): PDGFRβ (green) and CD31 (red) double immunolabeling was performed on CTL (**A**–**C**) and PILO 3D (**F**–**H**) rats. Similarly, ColIV (green) immunolabeling was performed on CTL (**D**,**E**) and PILO 3D rats (**I**–**J**). Cell nuclei were counterstained with DAPI (blue) (**C**,**E**,**H**,**J**). CC, corpus callosum; O, stratum oriens; P, pyramidal neurons of CA1, CA2, and CA3; R, stratum radiatum; LM, stratum lacunosum-moleculare; M, molecular layer; DG, dentate gyrus; G, granule cell layer of the DG; H, hilus of the DG. PDGFRβ, CD31, and ColIV are markers of pericytes, endothelial cells, and BM, respectively. PDGFRβ staining was detected in the hippocampi of CTL rats (**A**,**C**) and increased in PILO 3D rats, particularly in and around blood vessels (**F**,**H**). CD31 staining was concentrated in blood vessels of the LM (**B**,**C**) in CTL animals and increased significantly in LM blood vessels as well as throughout O, P, and R in PILO 3D rats (**G**,**H**). Weak ColIV staining was observed in the CTL rat hippocampi, in particular, in blood vessels of the LM (**D**,**E**), whereas prominent labeling of the vasculature was noted in PILO 3D rats, in O, P, R, LM, M, and DG (**I**,**J**). Scale bars: 250 μm. Quantification of increased PDGFRβ, CD31, and ColIV proteins in rat blood vessels following PILO-SE. Histograms showing average percentage of the fluorescence intensity of these 3 markers in DG and LM vessels in CTL and PILO 3D rats (**K**). Blood vessels of PILO 3D rats expressed significant PDGFRβ, CD31, and ColIV levels compared to CTL vessels. PDGFRβ, CD31, and CollV levels were increased compared to CTL. Values are given as the mean ± SEM as a percentage of CTL. Asterisks indicate statistically significant differences: *** *p* < 0.001 (Student’s *t*-test).

We next analyzed the regulation of the BBB protein markers, using qualitative and semi-quantitative immunohistochemistry with PDGFRβ, CD31, and Collagen IV (ColIV) antibodies to assess correlations with the RT-qPCR BBB markers and follow their distribution and expression intensity at the cellular level of different areas of the hippocampus. Based on the RT-qPCR results showing peaks in the neuroinflammation markers GFAP and Iba1 during the latent period between PILO 3D and 7D, we conducted our immunohistochemistry analysis at PILO 3D. We first performed dual PDGFRβ and CD31 immunohisto-labeling on the CTL and PILO 3D rats (Figure 2A–C,F–H). At low magnification, in all areas and layers of the hippocampi of the CTL rats, weak to moderate PDGFRβ staining was observed in the nervous tissue parenchyma cells (Figure 2A,C). Note that numerous cells were also stained in the subgranular zone of the DG, both in the CTL and PILO 3D rats. In addition, moderate PDGFRβ expression was also observed in blood vessels of all sizes and shapes (see insets in Figure 2A,C). PDGFRβ and CD31 staining were increased in all hippocampal areas and layers of the PILO 3D rats, as shown by mosaic tile scans (Figure 2F,H). Indeed, increased PDGFRβ was observed in the oriens (O), pyramidal layer (P), radiatum (R), lacunosum-moleculare (LM), molecular layer (M), and in the dentate gyrus (DG). In the CTL rats, immunohistochemical analysis of rat brain sections showed CD31 immunoreactivity in blood vessels of all sizes and shapes (Figure 2B,C), which increased in PILO 3D LM blood vessels, as well as in the CC (corpus callosum), O, P, and R (Figure 2G,H). 

In the same line, we performed ColIV immunolabeling on the CTL and PILO 3D rats (Figure 2D,E,I,J). In the CTL rats, ColIV staining was observed only in the M of the DG lower blade (Figure 2D,E), whereas the PILO 3D rats displayed strong labeling of the vasculature in the O, P, R, LM, and M of the DG (Figure 2I,J). We refined our study by conducting semi-quantitative analysis on the CTL and PILO 3D rats, to determine the extent of changes in the fluorescence intensity of the different vascular markers (Figure 2K). In PILO 3D animals (PDGFRβ, 27 vessels; CD31, 53 vessels; ColIV, 21 vessels), the expression of all vascular markers increased significantly in the blood vessels of the DG and the LM compared to CTL animals (PDGFRβ, 15 vessels; CD31, 29 vessels; ColIV, 15 vessels). Specifically, PDGFRβ, CD31, and ColIV increased 2.4-, ~2- and ~1.9-fold, respectively (Figure 2K, *p* < 0.001, Student’s *t*-test). 

**Figure 3:**
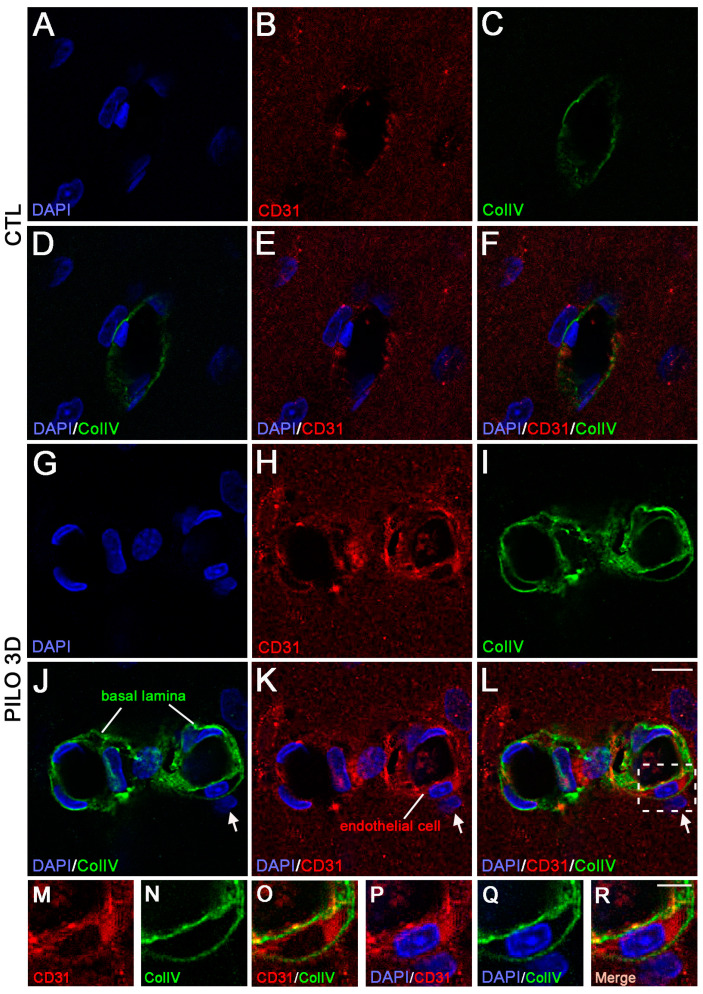
Double immunostaining followed by high magnification of LM blood vessels showed higher immunolabeling of CD31 (red) and ColIV (green) in PILO 3D rats (**G**–**L**) compared to CTL animals (**A**–**F**). Increased CD31 and ColIV occurred essentially within endothelial cells (red) and the basal lamina (green), respectively, as illustrated by higher magnification of the boxed-in area in (**L**) (**M**–**R**). Note that arrows in (**J**–**L**) point to a non endothelial cell. Cell nuclei were counterstained with DAPI (blue). Scale bars: 10 μm (**A**–**L**) and 5 μm (**M**–**R**).

**Figure 4:**
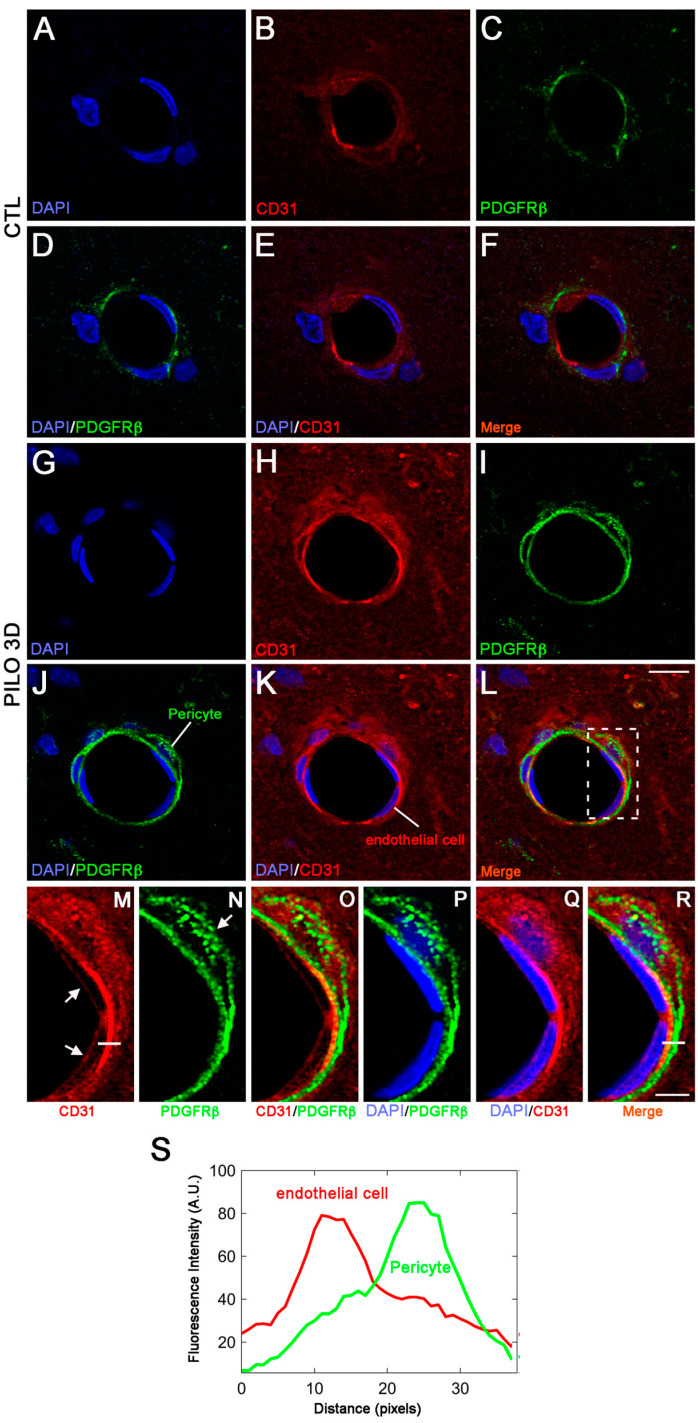
Double immunostaining followed by high magnification of LM blood vessels showed higher immunolabeling of CD31 (red) and PDGFRβ (green) in PILO 3D rats (**G**–**L**) compared to CTL animals (**A**–**F**). Increased CD31 and PDGFRβ occurred essentially within endothelial cells (red), and pericytes (green), respectively, as illustrated by higher magnification of the boxed-in area in (**L**) (**M**–**R**). Cell nuclei were counterstained with DAPI (blue). Arrows in (**M**) and (**N**) point to endothelial cells and pericytes respectively. Scale bars: 10 μm (**A**–**L**) and 5 μm (**M**–**R**). White lines in (**M**) and (**R**) insets are representative scans across the cell–cell borders. Fluorescence intensity profiles (arbitrary units, A.U.) of CD31 and PDGFRβ, in relation to distance (pixels), were obtained to determine whether these markers indeed stained endothelial cells (red line) and pericytes (green line), respectively (**S**).

We used high magnification to analyze in detail the double immunolabeling of CD31 (red) and ColIV (green) in CTL animals compared with the PILO 3D rats (Figure 3). The LM blood vessels displayed lower immunolabeling of CD31 (red) and ColIV (green) in CTL animals (A–F) when compared with the PILO 3D rats (G–L). Increased CD31 and ColIV immunolabeling appeared within endothelial cells (red), and the basal lamina (green), respectively, as illustrated by higher magnification of the boxed-in area in L (M–R). Indeed, ColIV immunostaining was closely associated with CD31 staining (Figure 3M–R), with basal lamina surrounding endothelial cells as reported elsewhere [13,34]. In conclusion, in rat PILO 3D hippocampi, CD31 and ColIV proteins were increased in rat blood vessel endothelial cells and basal membrane, respectively. Last, high magnification of CD31 (red) and PDGFRβ (green) double immunolabeling (Figure 4) shows that the LM blood vessels displayed lower CD31 and PDGFRβ expression in CTL animals (A–F) when compared with the PILO 3D rats (G–L). 

It has been shown that PDGFRβ pericytes surround endothelial cells and are found between the abluminal surface of the endothelial tube and the BM. Also, CD31 endothelial cells stain at the inner lining of blood vessels, while they are located between the BM and the abluminal surface of the endothelial tube [13,34]. When analyzed at high magnification, the boxed-in region in L showed endothelial cells (red) encircled by a pericyte (green) (Figure 4M–R). To confirm that indeed CD31 and PDGFRβ were expressed in distinct cell types, kymographs were constructed and analyzed with ImageJ 1.53 software from single-pixel width white lines taken from each channel of the confocal images (Figure 4K). Then, profiles of the signal intensities of CD31 (red line, K) and PDGFRβ (green line, K) were measured along the single-pixel width white lines drawn in (M) and (N), showing that immunolabeling of the two proteins is clearly distinct (Figure 4S). Thus, our results indicate that in PILO 3D, CD31 and PDGFRβ increased specifically in endothelial cells and pericytes, respectively. 

## 3. Discussion 

In the present work we investigated, during the different phases of epilepsy, in the rat hippocampus, the regulation of the neuroinflammatory GFAP and Iba1 markers and vascular proteins of the BBB, such as CD31, PDGFRβ, and ColIV. We used a well-characterized experimental model of TLE induced by PILO in adult rats and implemented RT-qPCR, qualitative and semi-quantitative immunohistochemistry, and confocal microscopy analysis. This PILO model was selected because it involves a dynamic reorganization of neuronal networks, gliosis, neuroinflammation, loss of integrity of the BBB, and neurovascular rearrangements in the hippocampus. This reorganization begins after the initial period of SE following PILO injection, during the silent period/latent phase when animals display normal behavior, and reaches a plateau at the chronic phase when the animals develop SRSs [3,4,5,35]. It is known that patients with epilepsy can experience respiratory alterations during seizures [36,37]. Indeed, seizures may cause apnoea that can reduce blood oxygen to a life-threatening level. Patients with epilepsy exhibit increased risk of myocardial infarction, stroke, and cardiovascular death [38]. In the PILO animal model, it is known that respiratory failure following convulsions is a common cause of acute death. Post-convulsive respiratory paralysis can be accompanied by hypothermia and by vascular congestion of the brain, heart, lungs, and kidneys [39]. More recently, it has been shown in rats with SE triggered by PILO that 30–50% of the epileptic rats exhibited a sharp decrease in oxygen consumption, a low metabolic rate of oxygen, and slow regular ventilation [40]. These percentages are consistent with 55 to 60% of animals surviving PILO administration, as reported by us and others [41,42,43]. 

In the PILO-treated rats, we used RT-qPCR to study the kinetics of mRNAs coding the GFAP and Iba1 reactive glial markers. We observed that mRNA levels of both markers significantly increased in the hippocampus during the latent phase and decreased during the chronic phase. These results agree with previous studies including ours [27,44,45,46] and confirmed neuroinflammation in our PILO rats. The upregulation of glial markers GFAP and Iba1 in the hippocampus was accompanied by increased CD31, PDGFRβ, and ColIVa1 mRNAs and concomitant protein expression during the latent phase (PILO 3D). These BBB markers decreased during the chronic phase (PILO 1M and/or 3M) when glial reactivity subsides. More specifically, the increase in CD31, PDGFRβ and ColIVa1 proteins occurred in cells of the BBB. These findings demonstrate that in our rat model of TLE, all three markers exhibited a significant increase, which may be indicative of structural alteration, remodeling of the BBB, or neuroprotection mechanisms. Our results question whether the three BBB markers whose expression is upregulated in our epilepsy model are beneficial or detrimental for vascular integrity. 

CD31 is mainly expressed by endothelial cells. This protein is a cell–cell adhesion and signaling molecule involved in angiogenesis and transmigration [47]. In agreement with our results, CD31 was induced in cerebral blood vessels between 24 and 72 h after Kainate administration in a mouse model of TLE [48]. It has been reported that peripheral and central inflammation promote breakdown of the BBB due to the upregulation of inflammatory mediators [49]. Hence, the increased expression of the vascular proteins that we observed may be involved in the breakdown of the BBB in PILO rats. However, BBB components may also be key players in the pathogenicity of TLE, independent of inflammation. For instance, the expression of CD31 by CNS endothelial cells is not required for initiation of inflammation and for the development of clinical signs in an animal model of multiple sclerosis [50]. CD31 maintains vascular integrity during inflammation by engaging with pro-survival pathways and by inhibiting cytokine production and pro-inflammatory signaling [8,51]. Accordingly, CD31 induction in our TLE model may be associated with anti-inflammatory and neuroprotection processes.

PDGFRβ is a marker of pericytes, which are spatially isolated contractile cells on capillaries that control cerebral blood low and BBB function in physiopathological conditions. Signaling through PDGFRβ regulates pericyte survival, proliferation, and migration. In TLE, we observed increased PDGFRβ expression in hippocampal blood vessels as expected, but also in nervous tissue parenchymal cells, suggestive of rearrangement in PDGFRβ distribution. The morphology and the distribution of these cells in all areas of the hippocampus, including in the CC, are reminiscent of glial cells. This observation agrees with Shen and colleagues [52] who detected PDGFRβ expression in cultured astrocytes isolated from neonatal mouse brain. Note that numerous cells were also stained in the subgranular zone of the DG both in the CTL and PILO 3D rats, suggesting that PDGFRβ may be expressed in newly formed granule cells of the DG. Consistent with this observation, PDGFRβ is reported to be expressed in some neurons [52,53,54,55]. Outside the CNS, PDGFRβ protein expression has been proposed as a rescue response to cardiac vascular pathological insult [56]. In agreement with our results, Klement et al. [15] recorded PDGFRβ mRNA increase in a mouse model of TLE induced by Kainate. In addition, within the regional scar that is a hallmark of epilepsy, a fibrotic-like PDGFRβ mesh was shown to develop around the capillaries, peaking at early stages post-SE, and regressing, but not resolving during the SRSs. Sakai and colleagues showed that the increased expression of PDGFRβ in the hippocampus after traumatic brain injury may lead to hypersensitivity to PILO in a relevant mouse model [57]. PDGFRβ was proposed as a possible pharmacological target in epilepsy and the PDGFRβ agonist PDGF-BB reduced mural cell loss, vascular pathology, and epileptiform activity [15,57,58]. In brain specimens of patients with TLE and hippocampal sclerosis (TLE-HS), increased perivascular PDGFRβ-positive pericytes and enlarged and tortuous vessels were observed compared to TLE-non-HS. Similarly, brain specimens derived from epileptic subjects affected by intractable seizures associated with focal cortical dysplasia displayed high perivascular PDGFRβ immunoreactivity—typical of pericytes—and revealed ramified PDGFRβ-positive cells proximal to microvessels [59]. The decreased expression of PDGFRβ from PILO 14D to PILO 3M may be due to the loss of mural pericytes known to occur in epilepsy [58]. Our results are also reminiscent of the expression of PDGFRβ in brain specimens of patients affected by drug-resistant epilepsy, where the increased expression and rearrangement of PDGFRβ labeling after SE suggests the involvement of pericytes in cerebrovascular modifications associated with epilepsy [11,60]. PDGFRβ labeling in animal experimental and human epileptic tissue is increased and appears to undergo distribution rearrangements in diseased tissue, associated with microvascular–pericyte–glia changes, scar formation, and inflammation. TNF-α, IL-6, and specially IL-1β promote such pericyte-related modifications. Notably IL-1β, shown to be deeply involved in the pathogenesis of epilepsy [11], alters pericyte morphology and facilitates the formation of pericyte–microglia aggregates in ex vivo hippocampal slices [15,61]. In our PILO model, we have previously shown that IL-1β is one of the most expressed cytokines [27]. It is thus likely that the changes we observe in hippocampal pericytes are mediated by this cytokine. These changes may also result from pericyte-glia scarring, where abnormal ColIII and ColIV accumulation or distribution have been associated with leaky capillaries during seizure progression [15]. 

ColIV is the most abundant component of the BM of endothelial and epithelial cells. In the brain, ColIV is produced predominantly by brain endothelial cells and pericytes and these cells are separated by a BM. Six ColIV alpha chains (ColIV4a1 to ColIV4a6) have been identified. We specifically studied ColIVa1 and ColIVa3, which are involved in brain vascular integrity [16]. Increased ColIV has been reported in brain blood vessels in mouse, rat, and sheep animal models and human stroke tissue [62]. ColIV induction was shown inside fibrotic scars [63], and following spinal cord injury in rats, where it may participate in glial scar formation [64]. An association has also been shown between seizures and the deposition of collagen in porcine brain through taenia solium neurocysticercosis [63]. ColIV was increased in other models of pathology around the microvasculature in rat chronic hypertension [65], and ColIV expression has been shown in brains of TLE patients, albeit outside the vasculature and in meninges [66]. We thus find some discrepancies on vascular vs perivascular ColIV distribution, considering that at the time points we studied, ColIV labeling was predominantly vascular in rat TLE. Interestingly, in epilepsy, collagen has been shown to have migrational properties, both in vitro and in vivo, on cells of the DG layer [67]. Since collagen is involved in scar formation typically associated with experimentally induced epilepsy [68], one can hypothesize that the increased ColIV that we observe in epileptic hippocampi is involved in TLE-associated scar formation, but also in the migration of cells, in particular, dentate granule neurons that play a critical role in SRSs. 

In conclusion, our data show that in brain diseases such as TLE with obvious reactive glial inflammation, glial scar formation, neuronal network reorganization, and BBB dysfunction, several BBB proteins such as CD31, PDGFR, and ColIV are increased. Although the RT-qPCR results were obtained using whole hippocampi, which include BBB but also brain parenchyma cells, we found good correlations in the expression levels using semi-quantitative IHC as an alternative approach. Besides protein expression levels, this approach yielded precious additional information at the level of the blood vessels and relative protein distribution in identified cell types, which other methods such as Western blot or ELISA cannot provide. This study has some limitations. The proteins we studied have been evaluated in other pathological conditions, at different time points, and in different species. It is thus difficult at this point to reach a consensus regarding the molecular and cellular processes at stake and to infer whether the observed changes at the molecular and cellular levels are detrimental or beneficial for BBB and brain parenchyma homeostasis. Many more components that control BBB pathophysiological properties need to be studied, including components of the neurovascular unit such as extracellular matrix proteins, tight junction associated proteins, etc. Some general principles are starting to emerge, raising the hope that a better understanding of BBB dysfunction in CNS diseases will help towards characterization of the mechanisms that control vascular integrity, the basis of BBB dysfunction during epilepsy, and the development of new therapeutic strategies.

## 4. Materials and Methods

### 4.1. Ethics Statement 

All experimental procedures involving rats were approved by National and European regulations (EU directive no. 2010/63) and agreed with the authorization for animal experimentation granted to the laboratory by the Préfecture des Bouches du Rhône (permit number: D 13 055 08) and to the project (no. 00757.02) by the French Ministry of Research and Local Ethics Committee.

### 4.2. Rat Pilocarpine TLE Model

Adult male Wistar rats weighing 200–290 g (n = 40, Charles River, France) were housed in an animal facility at 22–25 °C under a 12 h light/dark cycle, with free access to food and water. Rats were injected intraperitoneally (i.p) with a low dose of the cholinergic antagonist scopolamine methyl nitrate (2 mg/kg; Sigma, St. Louis, MO, USA) to minimize the peripheral effects of pilocarpine hydrochloride (PILO) (320 mg/kg; Sigma), a muscarinic cholinergic agonist diluted in 0.9% NaCl and administered i.p. 30 min after scopolamine. At 1 h after the onset of SE, animals received diazepam (10 mg/kg, i.p.), and thereafter were carefully monitored to ensure a high survival rate. Only animals that developed sustained SE after PILO injection were included in this study. Only seizures of grade 3 or greater on the Racine [69] scale were scored (i.e., forelimb clonus ± rearing ± falling). During the latent stage (PILO 3D–14D), the animals recovered from SE and appeared normal regarding food and water intake, weight, and body temperature, while they did not develop seizures. During the chronic stage (PILO 1M and 3M), the animals developed SRSs. The onset was 2–3 weeks for PILO rats as previously described [70]. The frequency and intensity of seizures differed to some extent from one animal to another, which is inherent to the model. In general, animals displayed an average of 3–4 seizures per day, similar to previous reports by our laboratory [41] and others [71]. A total of 30 rats were administered with PILO, 8 animals died following cardiac and respiratory failure as previously described [39,40], 3 did not develop SE, and 1 did not develop SRSs. Additionally, 4 animals did not reach the qualitative controls after fixation and brain observation (poor fixation or complete hippocampal sclerosis). Hence, a total of 18 PILO animals were included in the study, representing 55 to 60% as reported in other studies [42,43]. Of those, 6 control rats received an injection of 0.9% NaCl. Each group of PILO-treated rats was compared to saline-treated animals used as CTL. PILO-treated animals were studied at several post-injection intervals during the latent and chronic stages, using 2 semi-quantitative approaches to follow the regulation of expression of BBB markers and/or cellular expression and distribution. First, we used RT-qPCR, and second, we correlated with qualitative and semi-quantitative IHC. RT-qPCR yielded global results at the level of the whole hippocampus, while semi-quantitative IHC allowed us to follow the distribution and the expression intensity of BBB markers at the cellular level of different areas of the hippocampus, according to standard principles and approaches [72,73]. 

### 4.3. RNA Extraction and RT-qPCR

Hippocampal tissue from CTL and PILO-treated rats was lysed using Qiazol lysis reagent (Qiagen France, Les Ulis, France) and total RNA was extracted using the RNeasy Plus Universal Mini Kit (Qiagen) according to the manufacturer’s protocol. cDNA was synthesized from 500 ng of total RNA using the High-Capacity RNA-to-cDNAc Kit (Applied Biosystems, Waltham, MA, USA). We used 12.5 ng of cDNA for the RT-qPCR experiments which were performed as described in [27]. We used the 7500 Fast Real Time PCR System (Applied Biosystems), TaqMan Fast Universal PCR Mix (Applied Biosystems), and TaqMan Assays probes (see Table 1), which were validated and purchased from Applied Biosystems. These probes were compliant with MIQE guidelines as they guarantee high specificity, sensibility, reproducibility, and a high level of quantitative analysis.

The samples were run in duplicate on 96-well plates and analyzed with 7500 v2.0 software (Applied Biosystems). The conditions of the thermal cycle were as follows: initial denaturation at 95 °C, followed by 40 cycles of denaturation at 95 °C, hybridization and extension at 60 °C. Relative expression levels were determined according to the ΔΔCt (Ct: cycle threshold) method where the expression level of the mRNA of interest is given by 2-ΔΔCT, where ΔΔCT = ΔCt target mRNA − ΔCt reference mRNA (RPL13, Ribosomal Protein L13, was used as internal mRNA reference) in the same sample. 

For the Collagen RT-qPCR experiments, we selected primers that detect the ColIVa1 and ColIVa3 mRNAs encoding the vascular isoforms of ColIV. The results were presented as fold induction compared to control values, considered as being 1, and are presented as means ± SEM from 3 different rats (n = 6 hippocampi).

### 4.4. Tissue Preparation for Immunohistochemistry

Rats were deeply anesthetized through pentobarbital sodium injection (Nembutal, 120 mg/kg i.p., Ceva, France) and perfused through the heart with 0.9% NaCl followed with a fixative paraformaldehyde-based solution (Antigenfix, Diapath, Martinengo, Italy). Following induction of SE, animals were subjected to a standard perfusion fixation protocol with NaCl, followed by Antigenfix. The brains were removed 30 min later and were postfixed for 24 h at 4 °C in the same fixative solution. Finally, brains were rinsed 3 times in phosphate buffer (PB, 0.12 M, pH 7.2–7.4) and cryoprotected in 30% sucrose solution (Sigma) in PB 0.12 M until fully dehydrated. Brains were frozen in isopentane solution (Sigma) at −80 °C, fixed in O.C.T. Tissue Tek (Sakura Finetek, Torrance, CA, USA), and sectioned coronally at 40 μm with a cryostat. The sections were collected sequentially in wells of culture plates containing an ethylene glycol-based cryoprotective solution and stored at −20 °C until processing. Selected sections from each rat, covering the dorsal hippocampus (from bregma −3.30 to −4.80 mm) according to the Rat Brain Atlas [74], were used for immunohistochemical staining. Free-floating sections from CTL and PILO rats were always processed in parallel.

### 4.5. Immunohistochemistry

Free-floating sections were double-stained with anti-CD31 (PECAM-1; mouse, Abcam ab64543, 1:100, Cambridge, UK) and either PDGFRβ (rabbit, Abcam ab32570, 1:100) or Collagen IV (ColIV; goat, SouthernBiotech 1340-01, 1:250, Birmingham, AL, USA). The ColIV antibody detects ColIVa1 to ColIVa6 (according to SouthernBiotech), and among these isoforms, ColIVa1 and ColIVa3 are expressed in brain vascular tissue (see https://brainrnaseq.org, URL accessed 23 August 2023). Sections were permeabilized and saturated with PB 0.12 M containing 3% Bovine Serum Albumin (BSA) and 0.3% Triton X-100 in PB 0.12 M for 1 h at RT. They were stained with the first primary antibody in PB 0.12 M, 3% BSA overnight at 4 °C. The next day, sections were washed 3× in PB 0.12 M, stained with the appropriate second primary antibody, and washed 3× in PB 0.12 M. The ColIV and CD31 labeling were visualized with a donkey anti-goat AlexaFluor A488 and donkey anti-mouse AlexaFluor A594, respectively (both at 1:800; Thermo Fisher Scientific, Waltham, MA, USA), for 2 h at RT, in the dark. The PDGFRβ immunolabeling was visualized with a goat anti-rabbit biotin (1:200) for 2 h at RT followed by streptavidin AlexaFluor A488 (1:800; both from Jackson Immunoresearch, West Grove, PA, USA). Tissue sections were washed 3× in PB 0.12 M, then counterstained with with 5 mg/mL 4′,6-Diamidino-2-phenylindole (DAPI, Sigma) for 30 min at RT, in the dark. After 3 washes in PB 0.12 M, floating sections were mounted on Superfrost Plus glass slides using Fluoromount-G Mounting Medium (Thermo Fisher Scientific) and stored at −20 °C until imaging and analysis. Immunohistochemical controls for double-labeling experiments included incubation of some sections in a mixture of a primary antibody and normal IgG (mouse/goat/rabbit normal IgG). In all cases, these sections exhibited the same pattern of immunolabeling as sections processed for single labeling. Methods are described in [27]. 

### 4.6. Confocal Microscopy and Quantification

All cytochemical quantifications were performed blindly. Acquisitions were performed using a confocal laser-scanning LSM 700 Zeiss microscope with a ×20 or ×40 oil objective, and analysis of immunostaining images was performed using ZEN 212 SP5 software (Zeiss, Jena, Germany). The mosaic function was used to visualize the whole hippocampus. Z-stack function was also useful to determine precisely the co-expression of two markers in the same cells. Finally, ImageJ 1.53 software was used to quantify each immunolabeling. Pictures were binarized to 16-bit black and white images and a fixed intensity threshold was applied, defining each staining. Images were obtained with double averaging, frame size 1024 × 1024, and 1 AU pinhole for each channel. Quantification of blood vessel fluorescence intensity was obtained from at least 15 stained blood vessels of the DG and the LM from 2 sections per animal and on both hippocampi. This fluorescence intensity was measured by drawing and measuring around the inner and outer surface of various differently sized and shaped vessels and arterioles that were clearly identified morphologically. The mean fluorescence intensity in and around blood vessels was assessed using ImageJ 1.53 software for the 3 markers, as described in [23], from 3 CTL and 3 PILO rats at PILO 3D. Background fluorescence was set in the SR, in areas devoid of brain vessels in the same sections and was subtracted. An average value of 3 such areas was obtained from every image. Data are presented as the average fluorescence intensity of PDGFRβ, CD31, or ColIV, relative to CTL (percentage). Images were processed using Adobe Photoshop 3 software. Using ImageJ 1.53, the fluorescence intensity profiles (arbitrary units, A.U.), as a function of distance (pixels) (as described in [75,76,77]), were obtained for PDGFRβ and CD31 to determine whether the different markers stained distinct cell types of the BBB. 

### 4.7. Statistical Analysis

Sample sizes and statistical power were determined according to Dell et al. [78], and Festing and Altman [79], using the Biosta TGV sofware (https://biostatgv.sentiweb.fr). All experiments were performed on 3 rats per group. One-way ANOVA analysis followed by Dunnett’s post hoc test was used to compare the mean of each group with the mean of a CTL group (as used in [80]). Unpaired Student’s *t*-test was used to compare 2 independent groups (as used in [81]). All data are expressed as the mean ± SEM. Statistical significance was set at *p* < 0.05. Histograms and statistical analyses were performed with GraphPad Prism 5.0 statistical software.

## Figures and Tables

**Table 1:** Rat TaqMan probes used for qPCR analysis.

Gene Name	Gene Description	Probe ID
*Gfap*	*Glial fibrillary acid protein*	Rn01253033
*Iba1*	*Ionized calcium binding adaptor molecule1*	Rn00574125
*CD31 or PECAM-1*	*EndoCAM or Platelet endothelial cell adhesion molecule-1*	Rn01467262
*PDGFRβ*	*Platelet-derived growth factor beta*	Rn01502596
*ColIV a1*	*Collagen, type IV, a1*	Rn01482927
*ColIV a3*	*Collagen, type IV, a3*	Rn01400991
*RPL13*	*Ribosomal Protein L13*	Rn00821258

## Data Availability

Data is contained within the article.
